# The effect of counting duration on quantitative fecal egg count test performance

**DOI:** 10.1016/j.vpoa.2019.100020

**Published:** 2019-11-18

**Authors:** Megan Slusarewicz, Paul Slusarewicz, Martin K. Nielsen

**Affiliations:** a MEP Equine Solutions, 3905 English Oak Circle, Lexington, KY 40514, USA; b M.H. Gluck Equine Research Center, Department of Veterinary Science, University of Kentucky, 1400 Nicholasville Road, Lexington, KY 40546, USA

**Keywords:** Fecal egg count, FEC, Duration, McMaster, Automated

## Abstract

Fecal egg counts are the primary diagnostic tools of equine parasitology and use of the McMaster test and its variants in clinical practice is widely recommended. Manual counting is, however, prone to various sources of human error. For example, in real-world situations analysts can be under significant pressure to process high numbers of samples in a limited time. This practice could affect test result quality, but yet no studies have determined whether this is the case. This study’s purpose was to assess the effect of shortened counting duration (from either restricting counting time or counting only one grid of a slide) on McMaster test performance, and to compare the results to those of an automated test whose output is not subject to such limitations. Fifteen fecal samples from horses infected with strongylid parasites were divided equally into three groups based on high, medium and low levels of egg content (201–500, 501–1000 and 1001+ eggs/g). Slurries were produced from each sample and 10 subsamples of each were counted by both the McMaster and automated methods. McMaster slides were first counted at leisure, and then twice again with counting time being restricted to either one or two min. The effect of reducing sample processing time by counting only one grid of the McMaster slide was also assessed. Counting for one min significantly decreased manual egg counts by 50–60% relative to counts conducted at leisure (p < 0.001). While these decreases were somewhat ameliorated by counting for two min, the results were still approximately 10% lower than the at-leisure counts, a difference that was also statistically significant (p < 0.001). Furthermore, restricted counting duration also resulted in a significant decrease of approximately one-third in McMaster test precision, as assessed by the coefficients of variation (CoVs) of the 10 replicates of each sample, as did counting just a single grid of the McMaster slide. These differences effectively further improved the observed superior precision of the automated method compared to at-leisure manual McMaster counting, and the automated counts and their precision remained relatively unaffected following multiple analyses of the same processed samples. Taken together, these results indicate that analysists should carefully assess the possible effects on test performance of modifications to standard egg-counting procedures that are designed to account for real-world pressures, in order to achieve an optimal compromise between test accuracy and precision on one hand and practicality on the other.

## Highlights

• Rapid counting reduces McMaster accuracy by 50–60% and precision by one third.
• Counting only one McMaster grid does not affect accuracy but decreases precision by one third.
• Automated counting operates with equal accuracy to McMaster but twice the precision.
• Strongylid ova suspended in sodium nitrate become translucent over time.
• Increased translucency is associated with a 5% underestimation of egg counts.

## Graphical abstract

**Figure:**
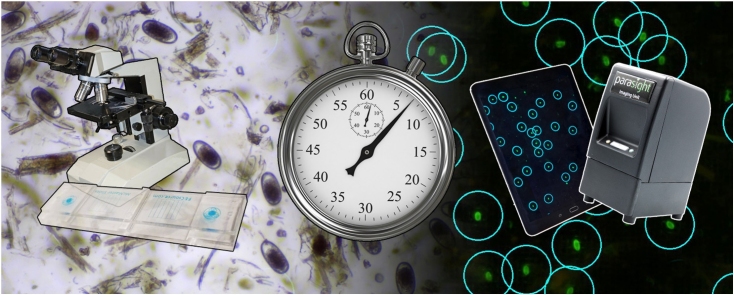

## 1 Introduction

The fecal egg count (FEC) is a primary diagnostic tool of veterinary parasitology, including in equines (Nielsen et al., 2018). Increasing levels of anthelmintic resistance in commonly occurring equine parasites (Peregrine et al., 2014) has necessitated the routine use of FECs as a monitoring tool for evaluating treatment efficacy and overall parasite egg shedding level (Kaplan and Nielsen, 2010, Nielsen et al., 2019, Rendle et al., 2019).

Numerous techniques exist for determining FECs, including the McMaster (Gordon and Whitlock, 1939), Stoll (Stoll, 1923), Wisconsin (Cox and Todd, 1962) and Mini-FLOTAC (Cringoli et al., 2017) methods. All of these are underpinned by a single principle, which is the separation of parasite ova from the bulk of the fecal detritus by flotation in a dense liquid medium, and subsequent microscopic examination and manual counting of the ova. The McMaster method is currently recommended for use in equine veterinary practice by the American Association of Equine Practitioners (AAEP) as a relatively simple and user-friendly method (Nielsen et al., 2019).

However, FECs are known to be highly variable (Carstensen et al., 2013) and we have hypothesized that a major source of variability is associated with inter- and intra-analyst variation during the manual counting process itself. We previously, therefore, developed an automated, filtration-based counting system, where an image of the sample is counted computationally by a specialized algorithm (Slusarewicz et al., 2016), that we hoped would eliminate the influence of human error in the counting process and thus decrease variability. In this study, we test our original hypotheses by examining the effect of one potential source of human error, *i.e.*, the time taken to count a McMaster slide, and by comparing the resulting counts to those produced with a commercial version of this technology.

In a busy veterinary practice or diagnostic laboratory, one possible contributing factor to egg count variability is technician workload, since a large number of samples (both fecal and non-fecal) may need to be completed within a given time frame. While many analyses can only be completed in a fixed period (*e.g.,* blood chemistry analyses), the time taken for egg counting can be foreshortened by evaluating slides more rapidly or counting one McMaster grid instead of the two present in a standard slide in order to accommodate the total workload. We hypothesized that such foreshortening, however, would have a negative effect on McMaster test performance (specifically on accuracy and precision) relative to the automated counting system, which, like a blood analyzer, produces objective results at a constant rate (once every 2.5 min).

The goals of this study were, therefore, to determine the effects of accelerated counting (by means of restricting counting duration or by counting only one grid) on McMaster FECs and to compare them to the automated egg counting system.

## 2 Materials and methods

### 2.1 Materials

The Parasight automated FEC system was provided by the manufacturer (MEP Equine Solutions, Lexington, Kentucky). FECA-MED 35.6% (w/w) sodium nitrate floatation medium (specific gravity 1.27) was from VEDCO Inc. (St. Joseph, Missouri, USA). Double-chamber McMaster slides were from FEC Source (Bank, Oregon, USA). A Nikon Eclipse E200 microscope was used for McMaster egg counting and an OMAX (Bucheon, South Korea) binocular compound microscope fitted with an MD500 camera (Amscope, Irvine, California, USA) was used for digital imaging in conjunction with Amscope 3.7 software. Microscope images were stitched together and annotated with Adobe Photoshop (Adobe Systems, San Jose, California, USA).

### 2.2 Samples

Samples were divided into three groups based on strongyle egg count in eggs per gram (EPG): Low (201-500 EPG), Medium (501-1000 EPG) and High (1001 or greater EPG). Lower (<200 EPG) egg count samples were not included in this study to avoid unnecessary interference of different detection limits on the precision estimates. Equine fecal samples obtained from the University of Kentucky’s research herd (Lyons et al., 1990) were screened using the McMaster method described in the AAEP parasitology guidelines (Nielsen et al., 2019) and by counting and averaging triplicate slides from the same fecal slurry. These results were used to assign each sample to each group (five individual samples per group). Each sample was coded to conceal the count and group assignment from the analyst.

### 2.3 Analysts

All samples were screened and classified according to their egg count level by the second author. The first author was thoroughly trained in the egg counting techniques, had four years of laboratory experience with these, and conducted all McMaster and automated counts reported herein.

### 2.4 Counting duration study

Subsamples of each fecal slurry were counted by both the McMaster and automated counting methods. To do this, the AAEP method was modified slightly with respect to the filtration step (which uses two layers of kitchen gauze). Since a critical step in the automated method is filtration through a series of filters prior to trapping the eggs on a fine mesh for staining and imaging, the cruder filtration of McMaster could potentially result in clogging of the terminal filter. As a result, samples for both tests were filtered through the sample preparation tool (SPT) of the automated system (Parasight; MEP Equine Solutions, Lexington, Kentucky, USA). Two SPTs were filled with 54 ml of FECA-MED and 6 g from a single fecal sample was added to each bottle and dispersed using the homogenizer built into the SPT. The samples were then filtered and pooled into a single Erlenmeyer flask, and this was used to fill ten McMaster slides (both chambers) and to run ten automated counts (4 ml per test) following the manufacturer’s instructions. Care was taken to resuspend the sample between each dispense by vigorous swirling.

Since manual McMaster counting involved multiple counts of the same slide for different time periods, the analyst was blinded to the identity of each slide between each set of ten counts. For this purpose, McMaster slides were coded on their undersides with numbered stickers that the analyst could not read prior to counting the slide, and after each count the analyst recorded the slide number along with the result. Slides were randomized by an individual other than the analysist between each set of 10 counts. Since the automated system is objective, no blinding was required between individual counts, although the analyst was blinded with respect to the identity of the sample.

The McMaster analyst conducted four counts on each slide at 100x magnification as follows. The analyst was permitted to conduct the first (long) count at their leisure, taking as much time as needed to perform a thorough examination of each slide (both grids). The time for each count was recorded along with the egg count and slide number. Next, the slides were randomized by an individual other than the analyst and the analyst counted each again, but this time was restricted to counting for one min. Counting duration control was achieved using an Android application (Talking Stopwatch v. 1.2.1; Robert Linsener, Google App Store), which provided an audible indication every six s (5 s × 12 grid lanes per slide = 60 s total). The identity of each slide was again recorded after each count. The slides were then randomized again and re-counted, but this time at a speed of two min per slide (10 s/lane). After a final randomization, the slides were subjected to a second full count at the analyst’s leisure. Slides were moved on and off the microscope with as much care as possible while trying to avoid tilting the slides in order to minimize the movement of ova during the course of the analysis.

Since counting time in the automated system is fixed, we instead assessed whether the time between sample processing and counting affected performance (since it is possible that an analyst may leave a sample unattended after processing but prior to counting while, for example, attending to another task). Thus, each sample was processed in series by the reagent dispensing system (2.5 min per test) and then imaged/counted. A second count of the same stained sample was made immediately afterward and then the filter was stored at ambient temperature. Each filter was then imaged/counted again 30 min after it was counted the second time so that there was a 30-min interval between the second and third counts of each sample. While the software in the automated system calculates the results in EPG by applying a multiplication factor (MF) of 4.6 to the number of eggs counted, McMaster counts were manually converted to EPG by applying a an MF of 33.3 (because of the ratio of sample to flotation medium used in the SPT) and the results rounded to the nearest whole number.

### 2.5 Single grid counting study

In this arm of the study, 6 g aliquots of the same 15 fecal samples were each suspended in 56 ml of FECA-MED in a single SPT and the slurry filtered through the tool and collected in a single flask. The individual samples were provided by an independent individual so that neither analyst was aware of their identities until the completion of their analyses. The slurry was aliquoted into 10 McMaster slides as described above, and each slide was then counted at the analyst’s leisure, with the count from each grid being recorded individually. The EPG values of the counts from each grid were calculated using an MF of 66.6 (for single-grid counts) or 33.3 (for the total from both grids).

### 2.6 Egg tracking and imaging

We had expected the first and second long McMaster counts to produce essentially the same results. However, preliminary experiments indicated a small, but reproducible decrease in egg count between these measurements (which represented a period of between two and three h). To investigate nature of this difference, we examined the fate of individual eggs suspended in flotation medium within McMaster slides.

To track the positions of eggs under McMaster slide grids during sample handling, enlarged schematic representations of grids were produced using Adobe Photoshop software, printed out, and used to record the position of each egg under each grid. Samples were prepared as with the other arms of the study but using a sample that was not used in the other experiments, and a total of four slides were assessed. The position of each egg was recorded on paper, and each slide was carefully handled between readings as described above. One h after the first slide was read, the process was repeated for each slide, with the analyst being allowed to use the first drawings for reference. The ova on the slides were then also re-mapped at 3 h.

For imaging, ova were first concentrated by centrifugation to maximize the number that could be visualized in a single field. A fecal sample (6 g) containing 2200 EPG of strongyle ova was processed with FECA-MED and a SPT as described above, and 30 ml of the pooled filtrate was divided between two 15 ml polycarbonate centrifuge tubes. The samples were spun for 5 min at 5000 x *g* in a fixed-angle benchtop centrifuge and the supernatants decanted, pooled and filtered through a 10 μm cell strainer (Pluriselect, Liepzig, Germany). The material on the surface of the strainer was recovered in 0.5 ml of FECA-MED using a pipette and dispensed into a McMaster chamber. After 5 min, three randomly selected, adjacent fields were photographed at 100x magnification. The sample was left in place in the microscope to minimize any egg movement and the three fields photographed at various times (1, 2, 3 and 5 h) using the same exposure times and gain settings. Images were composited using Adobe Photoshop.

### 2.7 Statistical analyses

The data were statistically analyzed using Statistical Analysis System (SAS) version 9.3 software (Cary, North Carolina, USA). Three mixed model analyses were conducted using the mixed procedure in SAS with egg count (EPG) as outcome variable, and strongylid egg count level (High, Moderate, Low) and counting method as explanatory variables. Sample ID and egg count replicate were kept as random effects. In the first analysis, the effect of egg count duration restriction was analyzed with first long count, 60 s count, 120 s count, and second long count as the four different method categories. In the second analysis, the automated strongylid egg counts were analyzed as outcome variable with first count, second count, and 30-min count as method categories. Lastly, the single *versus* double grid strongylid egg counts were analyzed with first grid, second grid, and both grids as method categories. If counting method was statistically associated with the outcome, a Tukey’s pairwise comparison of least squared means was performed. Least-square linear correlations were calculated using Excel software (Microsoft, Seattle, WA, USA).

A second set of mixed model analyses were then conducted with CoV as outcome variable and strongylid egg count level (High, Moderate, Low) and counting method as explanatory variables. Sample ID was kept as a random effect. The analyses dealt with the same three data sets as outline above. A fourth CoV analysis was done with the McMaster and automated counts combined into one data set to allow comparisons of CoVs obtained between the two techniques. All results were interpreted at the 0.05 significance level.

## 3 Results

### 3.1 Egg tracking

**Fig. 1:**
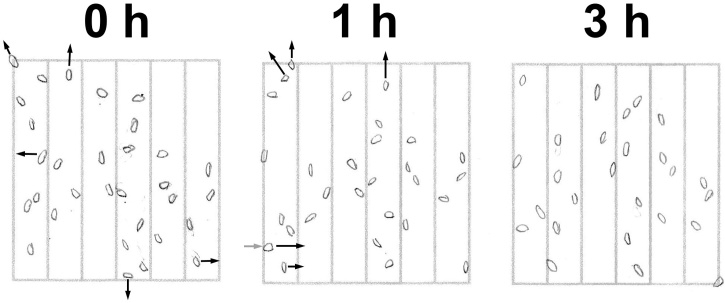
Mapping of egg positions in a McMaster chamber over time. Four McMaster slides were filled with fecal slurry in sodium nitrate flotation medium and the position of the eggs under each grid were mapped manually. Slides were removed from the microscope after each mapping and then replaced and re-mapped at one and three h. Maps of a representative grid at the various time points are shown. Black arrows represent eggs that will either move substantially or leave the grid at the next time point while the grey arrow represents an egg that entered the grid since the previous time point. Since the eggs were not observed continuously over the entire course of the experiment, assignments for these moments are tentative.

**Fig. 2:**
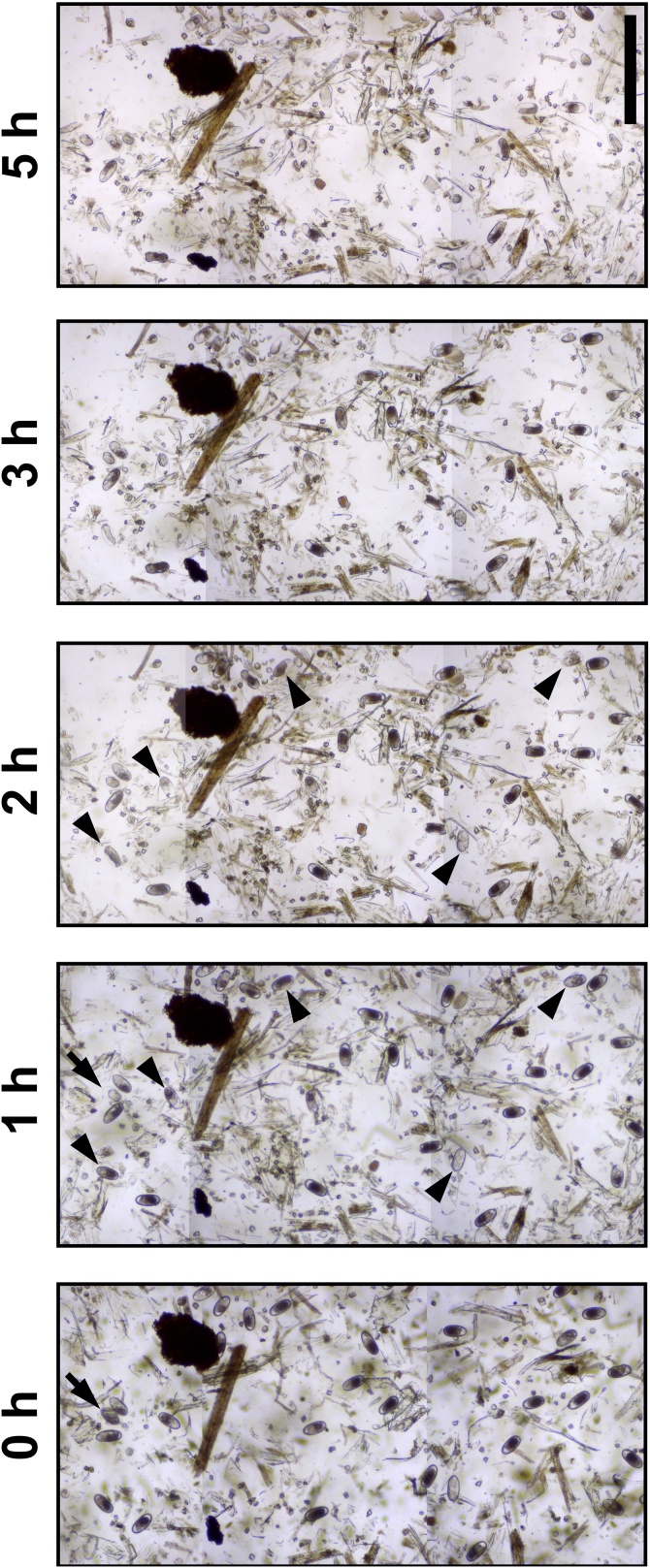
Morphological changes in ova in flotation medium over time. Centrifugally enriched ova were suspended in sodium nitrate flotation medium and placed in a McMaster slide. The slide was left undisturbed on the microscope stage and three adjacent fields were photographed at the given time points and used to produce the montages shown in the figure. Ova that became significantly translucent within 1 h are indicated by the arrows, while those that did so between 2 and 3 h are indicated by arrowheads. Bar =400 μm.

Pilot experiments for the counting duration arm of the study revealed a small, but reproducible, drop in the number of eggs counted in the second long McMaster count compared to the First. Manual tracking of egg positions showed that many underwent substantial shifts in their locations between counts, with some either leaving or entering the grid entirely. A representative series of maps from a single grid is shown in Fig. 1, where the number of eggs in the grid was 31, 29 and 26 at 0, 1 and 3 h respectively. Furthermore, simply sitting in flotation medium also led some eggs to undergo substantial changes in translucency (and therefore visibility) over time (Fig. 2). The rate at which this occurred varied for each egg, but one became translucent within one h (Fig. 2, arrows), while four others did so within two (Fig. 2, arrowheads). The majority of ova followed suit within three h and almost all within five. The increase in translucency was often accompanied by a shrinking of the cellular contents of the egg.

### 3.2 Counting duration

The duration of the first and second long counts averaged 4.1 min ± 0.65 and 3.8 m in. 0.47 (mean ± S.D.), respectively (Table 1). There were no significant differences between these counting times, nor those between the three egg level groups.

**Table 1:** McMaster counting times. The average counting times for the ten slides of each sample for both the first and second long counts are given in minutes.

	First Long Count			Second Long Count		
	H	M	L	H	M	L
	3.4	4.3	3.9	4.1	4.1	3.8
	5.5	3.9	4.1	5.1	3.6	3.6
	3.9	4.6	4.3	3.4	3.7	3.7
	3.4	5.0	3.6	3.5	4.0	3.5
	3.5	4.2	3.1	3.5	3.9	2.9
Mean	3.9	4.4	3.8	3.9	3.9	3.5
SD	0.9	0.4	0.5	0.7	0.2	0.3

Each number represents the mean from 10 independent counts from a single fecal slurry.

H = High egg burden group, M = Medium group, L = Low group, S.D. = standard deviation.

**Fig. 3:**
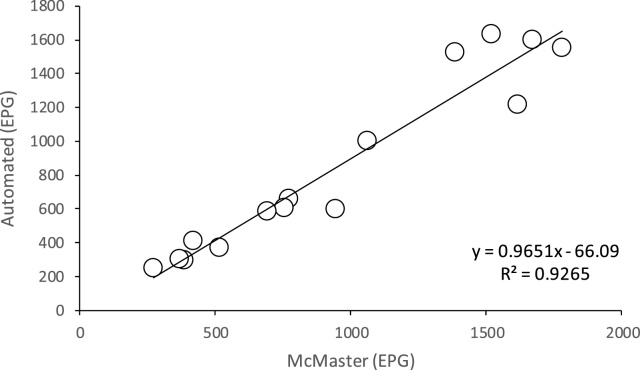
Correlation of manual McMaster and automated fecal egg counts. The average count in eggs per gram (EPG) of each set of ten replicates from the fifteen samples in the study obtained by both McMaster and automated methods were averaged and plotted against each other.

The averages of the ten replicates of the first long McMaster and first automated counts of each sample exhibited a strong positive linear correlation (R^2^ = 0.93; Fig. 3) with a regression coefficient of 0.97. The means of each set of ten replicates of each McMaster and automated count are presented in Table 2. The averages of High, Medium and Low group results for the first long McMaster and first automated counts data produced a straight line with an R^2^ of 0.99 and a regression coefficient of 0.99 (not shown).

**Table 2:** Absolute and relative strongylid egg counts obtained using manual McMaster and automated methods. All results presented as eggs per gram (EPG) of feces.

		McMaster							Automated				
		EPG				% of Long 1			EPG			% of Count 1	
		Long 1	1 min	2 min	Long 2	1 min	2 min	Long 2	Count 1	Count 2	Count 30	Count 2	Count 30
	High Group	1615	1062	1455	1485	65.8	90.1	92.0	1222	1209	1265	99.0	103.6
		1518	979	1332	1475	64.5	87.7	97.1	1642	1631	1647	99.3	100.3
		1778	776	1365	1605	43.6	76.8	90.3	1560	1579	1605	101.2	102.9
		1672	1285	1552	1608	76.9	92.8	96.2	1602	1606	1659	100.2	103.5
		1385	806	1126	1310	58.2	81.3	94.6	1530	1539	1608	100.6	105.1

	Medium Group	942	556	829	899	59.0	88.0	95.4	601	606	668	100.8	111.1
		773	473	706	759	61.2	91.4	98.3	665	655	683	98.6	102.8
		756	346	589	719	45.8	78.0	95.2	607	604	623	99.6	102.6
		693	303	536	676	43.8	77.4	97.6	590	581	571	98.6	96.9
		1062	513	932	1026	48.3	87.8	96.6	1007	1005	975	99.8	96.8

	Low Group	420	263	353	386	62.7	84.1	92.1	413	407	440	98.7	106.5
		386	190	316	350	49.1	81.9	90.5	301	307	321	102.1	106.6
		370	213	286	306	57.7	77.5	82.9	310	314	318	101.4	102.7
		270	190	260	290	70.4	96.3	107.4	251	250	262	99.8	104.5
		516	283	463	569	54.8	89.7	110.3	372	374	371	100.6	99.9

Mean	H	1594	982	1366	1497	61.8	85.7	94.0	1511	1513	1557	100.1	103.1
	M	845	438	719	816	51.6	84.5	96.6	694	690	704	99.5	102.0
	L	392	228	336	380	58.9	85.9	96.6	329	331	342	100.5	104.0

SD	H	149.7	207.4	159.3	122.2	12.2	6.6	2.9	167.2	173.1	164.8	0.9	1.7
	M	152.6	108.8	164.4	144.1	8.0	6.4	1.4	177.3	178.0	157.4	0.9	5.8
	L	89.0	43.0	79.1	112.3	8.1	7.3	11.7	63.5	61.3	66.8	1.3	2.8

Samples are categorized into three groups (High, Medium and Low) based on their egg burden.

Each number represents the mean from 10 independent counts from a single fecal slurry.

Long 1 and Long 2 are the first and second at-leisure manual counts, respectively.

Count 1 is the first automated count, Count 2 is the second automated count performed immediately after Count 1, and Count 30 is the final automated count performed 30 min after Count 2.

“% of Long 1” and “% of Count 1” refer to the relative change relative to the first count.

H = High egg burden group, M = Medium group, L = Low group, EPG = eggs per gram, S.D. = standard deviation.

The one-min McMaster counts generated values that were approximately 40–50% lower than those of the first long counts and these differences were all statistically significant (p < 0.0001). The count reductions were less dramatic when slides were examined for two min, but these were nevertheless 14.3–15.3% lower than the first long counts. The two-min counts were significantly higher than the one-min counts (p < 0.001), but still lower than both the first long (p < 0.001) and second long counts (p = 0.005).

In contrast, two sequential analyses of the same stained samples conducted within 1 min of each other by the automated method resulted in counts that were within 0.5% of each other with no statistically significant differences (p = 1.000). Counting the same sample 30 min later resulted in a small (2–4%) increase in the counts that was statistically significant relative to both the first (p = 0.039) and the second counts (p = 0.037).

The precision of both methods was assessed by analysis of the coefficients of variation (CoVs) generated from each set of ten replicate analyses of each sample (Table 3). The first long McMaster counts exhibited a trend of increasing CoV with decreasing sample egg count; however, these differences were not statistically significant. A similar trend was observed in the second long McMaster count, although in this case the difference between the High and Low groups was significant (p = 0.004). There were no significant differences between the CoVs of the first and second long counts of each group.

**Table 3:** Coefficients of variation (CoVs) obtained using manual McMaster and automated methods. Data presented by egg count level group (high, medium, low) and means and standard deviations (SD).

		McMaster				Automated		
		CoV				CoV		
		Long 1	1 min	2 min	Long 2	Count 1	Count 2	Count 30
High		13.0	18.2	19.4	15.1	8.0	9.0	9.6
		21.7	33.1	26.1	19.5	9.0	8.7	7.6
		16.8	33.4	21.0	17.4	8.2	6.6	9.4
		14.9	18.4	17.3	16.0	5.6	5.1	6.4
		21.0	25.6	27.6	20.1	8.7	7.5	7.8

Medium		12.2	34.1	19.0	13.9	10.4	10.3	11.8
		21.8	35.7	23.9	19.9	11.6	12.2	12.9
		15.8	33.1	21.0	14.7	8.4	8.0	11.4
		29.4	36.4	23.0	29.6	8.7	9.5	11.1
		20.3	28.3	20.0	20.4	7.7	8.2	9.2

Low		20.9	35.0	30.6	29.9	11.7	13.5	10.2
		32.8	31.0	33.7	29.2	12.6	15.7	18.2
		28.0	39.8	33.4	36.2	10.2	9.3	12.1
		18.8	34.1	30.1	23.6	11.8	10.1	12.7
		19.3	37.3	21.0	38.9	13.2	10.5	13.3
Mean	High	17.5	25.7	22.3	17.6	7.9	7.4	8.2
	Medium	19.9	33.5	21.4	19.7	9.3	9.6	11.3
	Low	24.0	35.5	29.8	31.6	11.9	11.8	13.3

SD	High	3.8	7.5	4.4	2.2	1.3	1.6	1.3
	Medium	6.5	3.2	2.1	6.3	1.6	1.7	1.3
	Low	6.2	3.3	5.2	6.1	1.1	2.7	3.0

Each number represents the coefficient of variance from 10 independent counts from a single fecal slurry.

Long 1 and Long 2 are the first and second at-leisure manual counts, respectively.

Count 1 is the first automated count, Count 2 is the second automated count performed immediately after Count 1, and Count 30 is the final automated count performed 30 min after Count 2.

The CoVs of the first automated counts were all significantly lower than both the first and second long manual counts of the corresponding McMaster samples (p*<*0.001). The automated system performed with significantly lower CoVs than the McMaster technique at all three egg count levels (p < 0.0001). Furthermore, the difference between the automated count CoVs of the High and Low egg count categories was statistically significant (p < 0.001), whereas the Medium category was not significantly different from the two other groups. The CoVs of the second automated counts were very similar to those of the first and exhibited no significant differences (p = 0.987).

Time-restricted one-min manual counting significantly increased the CoV relative to the first and second long counts (p < 0.001). The increase relative to the first long count was by 1.47-, 1.68- and 1.48-fold in the High, Medium and Low groups, respectively. Counting for 2 min resulted in CoV increases of 1.27-, 1.07- and 1.24-fold in the three respective groups, but these differences were not significantly different from the long counts. Increases of 1.04-, 1.21- and 1.12-fold were observed between the first automated counts and those conducted 30 min later, but none of these were statistically significant (p = 0.987).

### 3.3 Single grid counting

The results from counting of one and two grids on the McMaster chamber are presented in Table 4. There were no significant differences in the magnitudes of strongylid egg counts when counting one *versus* both grids of the McMaster chamber (p = 0.122). However, the counts generated from the first grid were significantly higher than those generated from the second (p < 0.001).

**Table 4:** Single- and double-grid McMaster counts given in eggs per gram (EPG) of feces and associated coefficients of variation (CoVs). Results are presented per egg count level group (High, Medium, Low) along with means and standard deviations (SD).

EPG												
	High Group				Medium Group				Low Group			
	Grid 1	Grid 2	(G1 + G2)/2	Range %	Grid 1	Grid 2	(G1 + G2)/2	Range %	Grid 1	Grid 2	(G1 + G2)/2	Range %
	1359	1132	1245	18.2	826	766	796	7.5	480	220	350	74.3
	1365	1265	1315	7.6	1232	906	1069	30.5	440	413	426	6.3
	1279	1192	1235	7.0	806	693	749	15.1	406	473	440	15.2
	1312	1166	1239	11.8	793	573	683	32.2	433	400	416	8.0
	1225	1006	1116	19.7	526	653	589	21.5	233	206	220	12.1
Mean	1308	1152	1230	12.9	836	718	777	21.4	398	342	370	23.2
SD	58.2	95.5	72.0	5.9	252.9	126.0	180.6	10.4	96.0	121.2	91.0	28.8

CoV												
	High Group				Medium Group				Low Group			
	Grid 1	Grid 2	(G1 + G2)/2	G1 + G2	Grid 1	Grid 2	(G1 + G2)/2	G1 + G2	Grid 1	Grid 2	(G1 + G2)/2	G1 + G2
	23.5	32.1	27.8	13.4	34.5	38.7	36.6	30.5	39.7	40.5	40.1	36.0
	25.3	30.3	27.8	23.4	16.6	34.9	25.7	21.8	50.1	44.9	47.5	39.6
	25.7	27.7	26.7	21.0	29.0	29.1	29.1	22.1	47.9	64.5	56.2	38.6
	23.8	19.8	21.8	14.1	37.9	54.3	46.1	23.4	28.3	44.4	36.4	16.5
	18.7	33.4	26.0	14.5	16.3	31.1	23.7	15.3	62.1	49.2	55.6	44.7
Mean	23.4	28.7	26.0	17.3	26.8	37.6	32.2	22.6	45.6	48.7	47.2	35.1
SD	2.8	5.4	2.5	4.6	10.0	10.0	9.2	5.4	12.6	9.4	8.9	10.8

Each number represents the mean or coefficient of variance from 10 independent counts from a single fecal slurry.

Grid 1 and Grid 2 represent results from counting only the left and right grids of the McMaster slide, respectively, while “G1 + G2” represents results from counting both grids.

CoVs were calculated for each pair of ten single-grid counts as well as for their sums (*i.e.*, double-grid counts, which were essentially a repeat of first long counts in the counting duration arm of the study on different subsamples of the same fecal material). The CoVs obtained from counts produced when considering both grids were consistent with those observed in the first and second long counts in the counting duration arm of the study (c.f. Table 3, Table 4). The average CoVs of the ten single grid counts in each group were 26.0%, 32.2% and 47.2% for the High, Medium and Low groups, respectively. These were 1.5-, 1.4- and 1.3-fold larger than the CoVs produced from counting both grids of each slide. Counting one grid alone resulted in significantly higher CoVs than counting both grids (p < 0.01).

## 4 Discussion

The goals of this study were to determine whether reducing the manual counting time of McMaster slides affects assay performance and to compare this performance to that of an automated system that eliminates variables generated by human subjectivity and error. Our results show that both accuracy and precision are significantly and adversely affected when one min is taken to count McMaster slides and to a lesser, yet still significant, extent when counting for two min. Furthermore, while reducing counting time by reading only one grid on the McMaster slide had no significant effect on accuracy, doing so significantly reduced precision relative to counting both grids of each slide.

The automated egg counting system evaluated in this study is based on the staining of chitin in helminth eggshells using a fluorescently derivatized recombinant chitin-binding protein (Slusarewicz et al., 2016). This labelling allows for the collection of high-contrast images of stained ova, which can then be counted by image processing software. The high contrast nature of the images means that ova can be identified at very low magnification, which facilitates the imaging of a large amount of fecal material in a single photograph (approximately 10 times more than that contained in the two grids of a McMaster slide). A reduction in the variability of stochastic sampling due to the larger sample size may partially explain why early prototypes of the automated system performed with greater precision than McMaster, although it was also superior to Mini-FLOTAC (Scare et al., 2017), which uses even more fecal material, suggesting that other factors may also be involved in this superior performance.

These factors may stem from the fact that the automated method counts ova objectively and so is unaffected by parameters such as the degree of analyst training/skill level with respect to correctly identifying ova and fecal debris; analyst fatigue (particularly after extended time at the microscope); modifications to McMaster techniques between laboratories that have not been validated with respect to their effect on the counts obtained; and the speed at which samples are counted. These sources of variability could potentially lead to discrepancies in counts between laboratories, and even within the same laboratory, yet surprisingly there have been no studies conducted to date to assess the magnitudes of their effects on test results. Interestingly, there were no significant differences in the time taken to count slides at leisure with respect to the different egg burden groups, suggesting that the number of ova in the sample did not subconsciously cause the analyst to modify their counting speed.

The second long counts in the counting speed arm of the study served as controls to demonstrate that any differences at one and two min were not due to a systematic loss/gain of eggs over time during the process of counting one sample, which took between two and three h. There was a reproducible loss of approximately 5% of the ova between the two long counts, which was significant in the High and Medium groups. We found that a proportion of eggs changed positions during this process, with some leaving and entering the grids entirely. This may have been due to the physical processes of placing the slides onto, and removing them, from the microscope stage between time points and/or due to the dynamic sinking and floating of eggs that has been observed over time (Norris et al., 2019). However, such stochastic repositioning of ova would be expected to, on average, produce variations in the second counts that centered around the means of the first. This was clearly not the case, since the second long counts were consistently smaller than the first.

During the mapping of ova onto grids, we observed that some had become translucent, particularly after 3 h in the McMaster slides. We investigated this phenomenon by producing time-lapse photographs of ova floating in sodium nitrate. This confirmed that the ova did indeed fade substantially with time, with some becoming more difficult to distinguish within one or two h, which is the approximate period that separated the two long counts. While the positions of some of the ova shifted slightly between time points, none disappeared or appeared, suggesting that sinking and/or refloating was not contributing to the reduction in the magnitudes of the second long counts. Furthermore, the lack of significant spatial movement of the ova as the slide was left stationary on the microscope stage suggests that the large positional shifts observed during mapping were due to the physical manipulation of slides as they were placed on and removed from the microscope. Taken together, these data suggest that the observed decline in counts was due to the fading of ova, which led to some being overlooked during the second count.

Although the mechanism underlying this phenomenon remains unclear, one possibility is that it due to an equilibration between the flotation medium and the inside of the egg, leading to a reduction in refractive index differences and thus a loss of contrast. This hypothesis is supported by the observed shrinkage of the cellular material in the eggs, presumably due to dehydration by osmosis. This effect may have consequences on the accuracy of counts conducted when multiple samples are prepared simultaneously and then counted sequentially since there may be significant reductions in sample counts between the first slide to the last if the period is longer than two h. Thus, analysts should consider minimizing the length of time in which ova are left to stand in McMaster slides in order to avoid this small, but reproducible, drop in egg counts. However, it should be noted that this study utilized a sodium nitrate flotation medium, and so further work will be required to determine whether this phenomenon also occurs with media of differing compositions. Furthermore, there appeared to be a spectrum of rates at which different ova became translucent, with some doing so within one or two h and others taking up to 5 h. The phenomena underlying these differences are unclear and require further investigation to determine whether they are due to, for example, differences in strongylid species, ova health/viability or other physical or physiological reasons.

The long manual counts obtained here corresponded well with those produced by the automated system, as evidenced by a correlation coefficient close to unity, which was to be expected since the latter has been calibrated to produce results that correspond to the former. However, the fall in the magnitude of the long counts over time represents a confounding factor when comparing differences between the different time-restricted counts. Nevertheless, compared to the first long count, there were statistically significant drops of approximately 50–60% and 15% in the one- and two-min counts, respectively, and these differences were also significantly different from the second long counts, which exhibited a drop of approximately 5%. Thus, the 15% drop observed after counting for two min was likely due, at least in part, to miscounting of the slides because of the imposed time constraint, and the count drop caused by increasing egg translucency was insufficient to mask this effect. These data therefore suggest that manually counting slides for less than two min can result in substantial underestimation of actual egg counts. Interestingly, this effect occurred at all three egg count levels, indicating that it was not due to the analyst overlooking eggs only when being overwhelmed by their sheer number, but rather that this was a systematic effect resulting from scanning through slides too rapidly.

While we could not vary counting speed for the automated test, back-to-back repeated counts by the automated method resulted in approximately 0.5% differences with no apparent directional preference. The automated system relies on a deep-learning algorithm that assigns probabilities to each particle in the image with respect to whether or not it is an ovum. Small differences between images, for example due to thermal noise, post-capture image processing and image compression, as well as slight movements of some of the eggs between images, may result in small changes to the likelihood estimations that, for some particles whose probabilities lie on the cusp of acceptance or rejection, can lead to a change in assignment and concomitant slight count variations between images. The automated counts increased slightly (2–4%), but significantly, after 30 min (which is the maximum post-staining period recommended by the manufacturer), but even this variation was substantially smaller than any of the changes observed in the manual counts. This increase may have been due to sample dehydration upon prolonged exposure to ambient atmosphere and subsequent changes to the appearance of the particles in the image, particularly with respect in increased contrast.

The data presented here indicate that the automated method operates with approximately twice the precision as an unrushed McMaster count performed by a well-trained analyst. This difference, however, was significantly affected by manual counting duration because while CoVs of the two long counts were essentially the same, they increased by 1.5-, 1.7- and 1.5-fold relative to the first long count in the High, Medium and Low groups, respectively, when slides were counted for one min; this effectively increased the relative precision of the automated method to approximately 3-fold that of McMaster at all egg count levels. These increases were lower with two-min counts at 1.3-, 1.1- and 1.2-fold respectively, with only those in the High and Low groups being statistically significant. Furthermore, counting one grid at leisure increased CoV (and therefore decreased precision) by approximately the same degree as counting both grids in one min (*i.e.,* 1.5-fold). This decrease in precision is likely due to the decreased sampling of the fecal slurry that is a result of examining the contents of only one grid. These data imply that, although both the McMaster and automated tests produce the same results when sufficient replicate analyses are performed on the same sample in order to average out sampling and experimental variation, the probability of any single result from a test being closer to the true egg content of the sample is substantially higher with the automated test than with the McMaster.

In summary, we have shown that duration-restricted counting of McMaster slides substantially decreases the accuracy of the count, with 40–50% fewer eggs being recorded when counts are conducted in one min. While two-min counts were substantially more accurate, they were still 10% lower than those conducted at the leisure of the analyst. In addition, duration-restricted counting (either by increasing counting speed or counting only one grid of the slide) also decreased the precision of manual McMaster by 1.5-fold. These simulations of real-world situations demonstrate the susceptibility of manual egg counts results to user-dependent variations that do not apply to the automated system.

Collectively, the findings from this study indicate that testing practices designed to increase the throughput of manual fecal egg counts result are a compromise between speed and a reduction in test accuracy and precision. These factors should be taken into account when considering methodological changes introduced to address practical concerns of sample throughput, particularly for tests that involve cognitively intensive tasks such as counting ova.

## Declaration of Competing Interest

PS is an employee and MKN and PS both hold stock in MEP Equine Solutions, LLC, which has filed two US patents for a commercially available automated image-based parasite egg counting technology.
